# Allostatic load and disordered white matter microstructure in overweight adults

**DOI:** 10.1038/s41598-018-34219-8

**Published:** 2018-10-26

**Authors:** J. Ottino-González, M. A. Jurado, I. García-García, B. Segura, I. Marqués-Iturria, M. J. Sender-Palacios, E. Tor, X. Prats-Soteras, X. Caldú, C. Junqué, O. Pasternak, M. Garolera

**Affiliations:** 1 Departament de Psicologia Clínica i Psicobiologia, Universitat de Barcelona, Barcelona, Spain; 2 Departament de Medicina, Universitat de Barcelona, Barcelona, Spain; 3 Institut de Neurociències, Universitat de Barcelona, Barcelona, Spain; 4 Institut de Recerca Sant Joan de Déu (IRSJD), Barcelona, Spain; 5 Institut d’Investigacions Biomèdiques August Pi i Sunyer (IDIBAPS), Barcelona, Spain; 6 CAP Terrassa Nord, Consorci Sanitari de Terrassa, Barcelona, Spain; 7 Unitat de Neuropsicologia, Hospital de Terrassa, Consorci Sanitari de Terrassa, Barcelona, Spain; 8 Brain, Cognition and Behavior Clinical Research Group, Consorci Sanitari de Terrassa, Barcelona, Spain; 9 Montreal Neurological Institute, McGill University, Montreal, QC Canada; 10 Departments of Psychiatry and Radiology, Brigham and Women’s Hospital, Harvard Medical School, Boston, MA USA

## Abstract

Overweight and stress are both related to brain structural abnormalities. The allostatic load model states that frequent disruption of homeostasis is inherently linked to oxidative stress and inflammatory responses that in turn can damage the brain. However, the effects of the allostatic load on the central nervous system remain largely unknown. The current study aimed to assess the relationship between the allostatic load and the composition of whole-brain white matter tracts in overweight subjects. Additionally, we have also tested for grey matter changes regarding allostatic load increase. Thirty-one overweight-to-obese adults and 21 lean controls participated in the study. Our results showed that overweight participants presented higher allostatic load indexes. Such increases correlated with lower fractional anisotropy in the inferior fronto-occipital fasciculi and the right anterior corona radiata, as well as with grey matter reductions in the left precentral gyrus, the left lateral occipital gyrus, and the right pars opercularis. These results suggest that an otherwise healthy overweight status is linked to long-term biological changes potentially harmful to the brain.

## Introduction

Overweight (body mass index [BMI] ≥25 kg/m^2^) is the result of sustained caloric surplus^1^. Anabolic (i.e., insulin) and catabolic signals (i.e., catecholamines, cortisol, and leptin) regulate energy reserves storage and expenditure via thermogenesis and lipolysis^2^. The abnormal accumulation of adipocytes during weight gain promotes the release of pro-inflammatory cytokines and increases the permeability of the blood-brain barrier (BBB) and the gastrointestinal tract, exposing the brain to harmful bacterial endotoxins and another inflammatory by products^3^. Ultimately, this can trigger physiological stress responses and disturb homeostasis through engaging the hypothalamic-pituitary-adrenal (HPA) axis^4^. Hence, overweight can precipitate long-term modifications in neuroendocrine and metabolic systems that subsequently hinder weight loss and may damage the brain. Cortical and subcortical structures are vulnerable to the effects of chronic stress^5,6^. Alterations in cortical top-down regulating areas (e.g., prefrontal cortex) and subcortical bottom-up processing regions (e.g., hypothalamus, basal ganglia, amygdala) have been proposed as an underlying mechanism in both stress^5^ and overeating^7^.

Stress is necessary for survival as it mobilises energy reserves (i.e., catabolic responses) to prepare us to overcome a threatening situation. Allostasis is the natural process whereby biological systems are pushed to their maximum capacity to guarantee fight-or-flight responses. However, frequent disruption of homeostasis can lead to a situation of allostatic load (AL). The AL is the ‘wear and tear’ of the body, and it has been associated with numerous comorbidities such as hypertension, type II diabetes, and dyslipidaemia^8,9^. Like chronic stress, the AL might jeopardise the integrity of the brain tissue by up-regulating cortisol and inflammatory cytokines release^6,10,11^. Nevertheless, only a few studies to date have assessed the relationship between AL and brain integrity^12–16^. Along with these works, this issue is similarly covered in chronic psychological stress or single stress biomarkers studies (e.g., cortisol, systolic arterial pressure). Likewise, chronic stress has also been proposed as a risk factor for obesity, as it plays an important role in feeding behaviour and body weight regulation. Cortisol enhances the desire for highly palatable food as a mechanism to restore the energy reserves consumed in surviving challenging contexts^17^. Equally, sustained discharge of stress hormones can also hinder weight control by affecting metabolic-related signalling (i.e., insulin and leptin resistance) responsible for thermogenesis and lipolysis processes^18,19^.

Diffusion tensor imaging (DTI) is a non-invasive neuroimaging technique that measures, *in vivo*, the displacement of water molecules in the brain^20^. Tract-based spatial statistics (TBSS) is the method of choice for assessing the microstructure of white matter (WM) tracts^21^. The most widely used WM scalar in TBSS is fractional anisotropy (FA), which measures the orientation dependence (from 0 to 1) of the water movement. If the tract is undamaged and well myelinated, motion along the fibre would be parallel to the fibre rather than perpendicular, returning higher FA values. However, decreased FA values are not specific to underlying pathological processes. In this vein, recent advances in DTI eliminate the effect of extracellular water content from surrounding brain tissue. This correction helps in discriminating processes that affect tissue, such as axonal degeneration, of processes that disturb the extracellular space, such as neuroinflammation^22^.

Obesity and TBSS studies have generally described inverse associations between waist circumference (WC) or BMI and FA in tracts involved in reward-seeking (e.g., inferior fronto-occipital fasciculi, corpus callosum, and corticospinal tract) and cognitive control (e.g., inferior and superior longitudinal fasciculi)^23–25^. Although contradictory findings do exist^26^, most indicate that the higher the BMI or the WC, the lower the FA^27–31^. In addition, studies on physiological markers of stress have found reductions in global and regional FA values^32–34^ linked to higher cortisol levels. Two other studies have reported negative associations between FA and cholesterol^35^ or high blood pressure^36^. Gianaros *et al*. (2013) found that the relationship between low FA values and low socioeconomic status was exacerbated not only by high levels of C-reactive protein but also by augmented adiposity. A decrease in this peripheral inflammatory biomarker was associated with higher FA values in a community-dwelling sample of older adults^37^. Aerobic fitness improves oxygen delivery to the brain, which promotes the expression of neurotrophic factors crucial in helping astrocytes to deal with the damaging outcomes of sustained stress and inflammatory responses. Overweight children^38^ and older adults^39^ presented higher FA values after participating in a physical exercise routine. Thus, these findings suggest that the cerebral WM has plastic properties: WM exhibits variations after recovering from chronic inflammatory states or engaging in healthy activities.

As mentioned above, an increase in AL (or chronic physiological stress) is related to an excessive engagement of the HPA axis, which can induce changes in food intake and fat storage^17–19^. Similarly, adipose-induced inflammation can activate the HPA axis by liberating pro-inflammatory cytokines^2,3^. Ultimately, an overly activated HPA axis may lead to adverse neurological outcomes, such as oxidative stress, decreased neurogenesis, or gliosis^6,10,11^. According to the AL model, overweight subjects could be enduring higher levels of stress compared to healthy weight participants. This could be detrimental to the brain as overweight is intrinsically linked to an excessively stimulated HPA axis and inflammatory responses. We explored this relationship in a previous study by conducting a cortical thickness analysis^15^. Overweight subjects presented higher AL indexes than lean participants. Moreover, the increase in AL in both groups was correlated with cortical changes of regions involved in regulating behaviour, reward processing, and controlling general cognitive function. Here we extend these findings on neuroanatomical differences and focus on microstructural white matter changes. Specifically, the present study is aimed at examining the relationship between AL and the composition of white matter tracts in participants with overweight and obesity. Several studies have correlated adiposity and physiological stress to changes in the microstructure of tracts involved in reward processing and cognitive performance. Additionally, we have complemented this principal analysis testing for variations in cortical grey matter (GM) morphology (i.e., volume). We expect to find augmented levels of AL in overweight subjects, as well as changes in the WM/GM composition regarding such increase.

## Results

Overweight and healthy-weight controls differed (p < 0.001) for all anthropometric measures (i.e., BMI, WC, and waist-to-height ratio [WtHR]) and the AL index. There were no statistical differences in the remaining sociodemographic, psychological and behavioural variables (Table 1).

**Table 1:** Overweight and lean participant’s variables of interest.

	Overweight (N = 31)		Lean (N = 21)	
	Mean (SD)	Range	Mean (SD)	Range
Age	31.12 (5.88)	21–40	29.95 (6.02)	21–39
Years education	13.58 (2.94)	9–20	14.48 (2.18)	10–18
IQ estimation	11.90 (2.21)	8–17	12.05 (1.86)	7–15
Female N (%)	19 (61%)		11 (52%)	
Smoker N (%)	8 (25%)		5 (23%)	
Drinker N (%)	14 (45%)		13 (61%)	
HADS anxiety	4.45 (2.78)	0–10	4.43 (2.52)	0–10
HADS depression	2.16 (2.19)	0–7	1.48 (1.60)	0–5
BMI	30.75 (4.86)	25.20–49.69	22.35 (2.01)	19.00–24.99
WC	96.97 (12.61)	82–137	78.56 (6.86)	68–92
WtHR	0.58 (0.07)	0.46–0.80	0.46 (0.03)	0.40–0.50
AL Index	6.64 (2.51)	3–11	3.38 (2.01)	0–7
**Frequency of family income in euros per month (%)**				
900–1,499	5 (16.1%)		4 (19%)	
1,500–2,099	14 (45.2%)		6 (28.6%)	
2,100–2,699	6 (19.4%)		3 (14.3%)	
>2,700	5 (16.1%)		7 (33.3%)	
Not available	1 (3.2%)		1 (4.8%)	
**Frequency of professional level (%)**				
Non-skilled	4 (12.9%)		1 (4.8%)	
Skilled manual	9 (29%)		4 (19%)	
Administrative	6 (19.4%)		5 (23.8%)	
Intermediate	4 (12.9%)		5 (23.8%)	
Professional	5 (16.1%)		3 (14.3%)	
Not available	3 (9.7%)		3 (14.3%)	

IQ estimation = intelligence quotient estimation (WAIS-III vocabulary scalar score), BMI = body mass index (kg/m^2^), WC = waist circumference (centimetres), WtHR = waist-to-height ratio (WC/height in centimetres) HADS = Hospital Anxiety and Depression Scale, SD = standard deviation.

**Figure 1:**
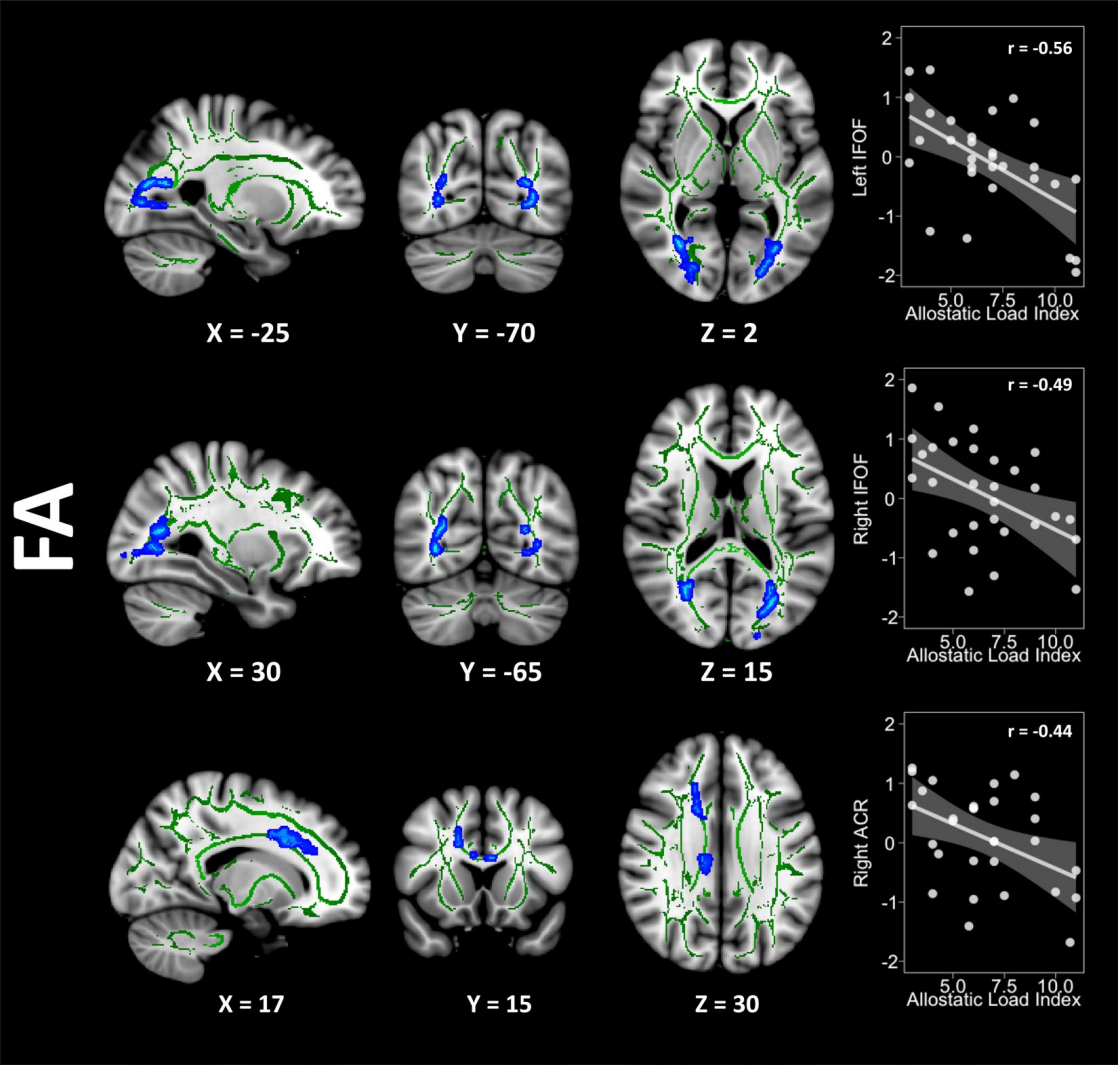
Decreasing FA values regarding the AL index increase are represented in blue. The Y-axis in the scatterplots depicts the standardised residual (regressors: age, sex, and WtHR) of the FA average score within the cluster. The X-axis represents the AL index scores. Please note that correlation coefficients only intend to complement scatterplots visualisation. FA = fractional anisotropy, WtHR = waist-to-height ratio, IFOF = inferior fronto-occipital fasciculus, ACR = anterior corona radiata.

The whole-brain comparisons between groups did not show differences between groups in any diffusivity scalar. Groups did not differ for the skeletonised maps either (see Appendix B1 in the Supplementary Information section). The overweight group showed negative correlations (family-wise error corrected p-value < 0.05) between FA values and the AL index in three clusters with their maximum intensity peaks located in the left inferior fronto-occipital fasciculus (IFOF) (X = −21, Y = −85, Z = 5, size = 473 voxels, p-corrected = 0.041), the right IFOF (X = 29, Y = −66, Z = 15, size = 349 voxels, p-corrected = 0.043), and the right anterior corona radiata (ACR) (X = 18, Y = 21, Z = 34, size = 342 voxels, p-corrected = 0.045). This group also showed a trend towards positive correlations (p = 0.09) between the AL index and the radial diffusivity (RD) and the mean diffusivity (MD) in clusters located in the right superior corona radiata and the body of the corpus callosum, respectively. There were no associations between the AL index and axial diffusivity (AD). Conversely, the lean group did not show either positive or negative correlations with any DTI metric. Cluster size, MNI coordinates, and extension of the FA results are available in Table 2. Figure 1 shows the cluster extension and the magnitude of the relationship between FA and AL index in the overweight group, depicted in a T1-weighted MNI template.

**Table 2:** Whole-brain correlations results in the overweight group.

	Peak	Voxels	MNI coordinates			Cluster extension	FWE peak p-value
			X	Y	Z		
FA	L IFOF	473	−21	−85	5	L anterior thalamic radiation, L cingulum (hippocampus), forceps major, L inferior fronto-occipital fasciculus, L inferior longitudinal fasciculus, L superior longitudinal fasciculus, L superior longitudinal fasciculus (temporal part)	0.041
	R IFOF	349	29	−66	15	R anterior thalamic radiation, R cingulum (hippocampus) forceps major, R inferior fronto-occipital fasciculus, R inferior longitudinal fasciculus and R superior longitudinal fasciculus	0.043
	R ACR	342	18	21	34	R anterior thalamic radiation, R cingulum (cingulate gyrus), forceps minor, R inferior fronto-occipital fasciculus, R superior longitudinal fasciculus and R superior longitudinal fasciculus (temporal part).	0.045

MNI = Montreal Neurological Institute, FWE = family-wise error, FA = fractional anisotropy, L IFOF = left inferior fronto-occipital fasciculus, R IFOF = right inferior fronto-occipital fasciculus, R ACR = right anterior corona radiate.

IQ estimation = intelligence quotient estimation (WAIS-III vocabulary scalar score), BMI = body mass index

(kg/m2), WC = waist circumference (centimetres), WtHR = waist-to-height ratio (WC/height in centimetres)

HADS = Hospital Anxiety and Depression Scale, SD = standard deviation.

**Figure 2:**
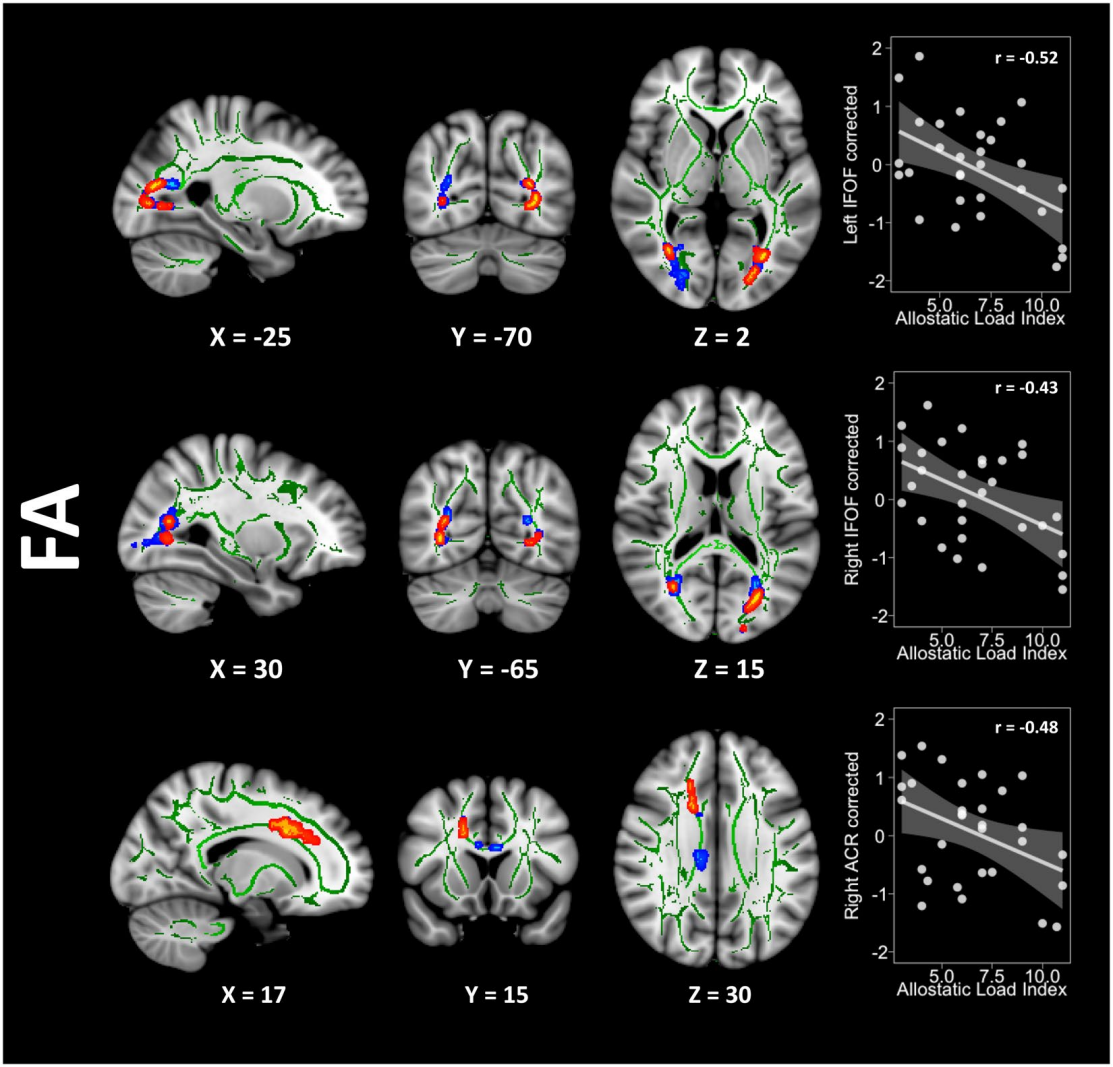
Overlapping results derived from the uncorrected (blue) and corrected (red) FA maps. The Y-axis in the scatterplots depicts the standardised residual (regressors: age, sex, and WtHR) of the FA average score within the cluster. The X-axis represents the AL index scores. Please note that correlation coefficients only intend to complement scatterplots visualisation. FA = fractional anisotropy, WtHR = waist-to-height ratio, IFOF = inferior fronto-occipital fasciculus, ACR = anterior corona radiata.

In an attempt to better characterise the pathological source behind this FA decrease, we conducted a *post-hoc* analysis with free-water (FW) corrected FA images in these clusters. Although significant, the results were less extended than the original ones (left IFOF size = 331 voxels, right IFOF size = 80 voxels, and right ACR size = 192 voxels). The comparison between uncorrected and corrected results is presented in Fig. 2. On another note, the FA results from the principal analysis vaguely extended to the corpus callosum after additionally controlling for tobacco smoking and non-pathological alcohol usage. In addition, FA results (as well as RD) were substantially more extensive when only controlling for age and sex, presumably because overweight subjects with low AL but high WtHR may not have driven the principal analysis results. However, since the aim of the study was to address the isolated relationship between AL and WM composition, we only discuss in depth the results of the principal analysis, leaving these available in the SI section (Appendix B2, C1, C2, D1).

**Figure 3:**
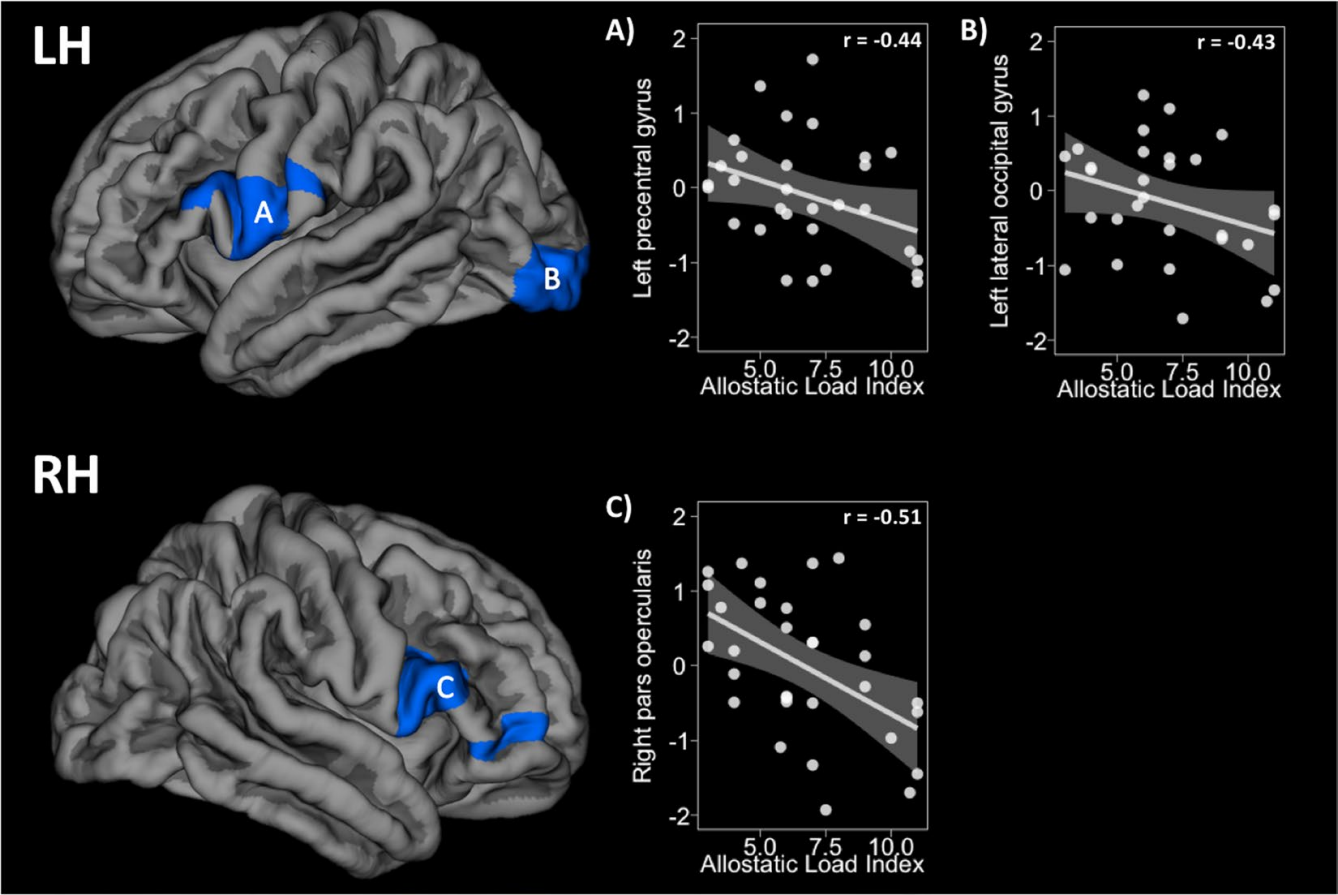
The first row shows the density reductions in the left hemisphere regarding AL increase in (**A**) the left precentral gyrus and (**B**) the left lateral occipital pole. The second row shows reductions in the (**C**) right pars opercularis. The Y-axis in the scatterplots depicts the standardised residual (regressors: age, sex, and WtHR) of the cluster average volume. The X-axis represents the AL index scores. Please note that correlation coefficients only intend to complement scatterplots visualisation. LH = left hemisphere, RH = right hemisphere, WtHR = waist-to-height ratio.

Moreover, overweight subjects showed volume reductions (cluster-wise corrected p-value < 0.05) regarding AL index increase in three clusters with their peak of maximum intensity in the left precentral gyrus (X = −60, Y = 2, Z = 14, size = 1756.31 mm^2^, Z-score = −2.20, p-corrected = 0.028), the left lateral occipital gyrus (X = −29, Y = −92, Z = −4, size = 1909.3 mm^2^, Z-score = −3.03, p-corrected = 0.015), and the right pars opercularis (X = 53, Y = 14, Z = 5, size = 2057.64 mm^2^, Z-score = −2.58, p-corrected = 0.010). Lean subjects did not show relationships between volume and AL index. Results of this analysis are shown below in Fig. 3.

## Discussion

In the present study, we examined the harmful effects that the relationship between chronic physiological stress (i.e., AL) and excess of weight might have on the brain. Here, we compared the microstructure of whole-brain WM tracts in relation to an AL index increase in two matched groups who only differed in anthropometric measures (i.e., BMI, WC, and WtHR) and did not present any cardiometabolic diagnosis (e.g., metabolic syndrome, type II diabetes). We found that overweight subjects presented higher AL indexes than lean controls. Our findings also showed that, regardless of the confounding effects of abdominal obesity (i.e., WtHR), the AL increase among the overweight participants was linked to an altered WM microstructure, as well as to changes in GM morphology.

We showed alterations in the WM composition relative to higher AL indexes in overweight subjects in the bilateral IFOF and the right ACR. Although the maximum peak of intensity was slightly asymmetrical between the right (Y = −66) and left (Y = −85) IFOF, the extension of both clusters comprised the same tracts. All three clusters spread to several projecting (i.e., corticospinal tract, anterior thalamic radiation, and corona radiata), commissural (i.e., forceps minor/major and corpus callosum), and associative tracts (i.e., cingulum and inferior/superior longitudinal fasciculi). Some of these tracts have also been described to be affected in overweight-to-obese subjects^24,25,27–29,40^ and chronic stress studies^32–36,41^. Even though a probabilistic tractography analysis would shed more light about this, these fibres anatomically connect mid-brain and superior cortical regions related to reward-seeking and supervising goal-directed behaviours^23–25^. Alterations in reward-seeking have been described in overweight^25^ and stress^42^ studies. A study from our group has also described an abnormal configuration of the reward-processing network^43^. In this study, obese participants also showed a disturbed composition (i.e., low FA) in tracts ascribed to such network (i.e., striatum, accumbens, and orbitofrontal cortex). Similarly, impairments in executive functions, such as inhibitory control and working memory, have previously been found in pathological caloric intake^44^ and stress^45^. Hypothetically, not being able of suppressing urgent drives (i.e., inhibitory control) or failing in predicting short-term consequences correctly (i.e., working memory) could lead to poor dietary choices and eating beyond caloric needs. Alarcón and colleagues (2016) have recently demonstrated that both left inferior-longitudinal fasciculus and left superior longitudinal fasciculus were inversely correlated to BMI, a relationship that mediated working memory performance^46^.

The less anisotropic diffusivity found in overweight volunteers linked to the AL increase may suggest underlying pathologies such as neuroinflammation, axonal degeneration, and/or structural remodeling^10,11,47–50^. To clarify this issue, we controlled for the contribution of FW within the clusters that emerged as statistically significant from the principal analysis. Microglia and astrocytes initiate inflammatory responses by prompting osmosis-inducing chemicals. This augments extracellular water content and subsequently affect water movement in adjacent functional tissue^51^. Yet axonal degeneration can be a process independent of extracellular water contamination, chronic inflammation can also affect oligodendrocytes and myelin sheaths, and therefore, escalate to long-term axonal degeneration^11,51^. In accordance with our hypothesis, the augmented levels of AL in overweight subjects may be linked to low-grade chronic inflammation states as well. When compared to the original results, the extension of the clusters substantially decreased (i.e., 30% in the left IFOF, 77% in the right IFOF, and 44% in the right ACR). It is immediately obvious that the right IFOF diffusivity was very influenced by the presence of interstitial water content, as 269 voxels vanished from this cluster after correction. Thereby, in the light of these results, and relative to normal-weight participants, overweight subjects endure higher amounts of physiological stress and sustained inflammatory responses that could have prompted axonal degeneration on a regional level, especially in the left IFOF and the right ACR.

None of the participants included in our study presented WM hyperintensities that could suggest neurological pathologies, which is often a missed factor in TBSS studies. Despite showing some advantages, this technique also presents limitations^21,52^. As an example of this, misalignments in registration are a common issue in TBSS. We dealt with this by registering our subjects onto a study-specific template. Moreover, signal dropouts and geometrical, or EPI distortions, are prone to occur along the phase-encoding direction at air-tissue interfaces such as the sinus, anatomically deforming the brain and worsening subject registration and alignment^53^. We overcame this by non-rigidly registering each DWI image to its corresponding T1-weighted sequence. This issue is something that the majority of TBSS studies with a single encoding-direction do not usually address, as additional sequences (e.g., field map, two-opposite acquiring directions) are mandatory. Additionally, ventricular CSF-contamination and extracellular water content may lead to underestimating DTI metrics, raising fair concerns about the nature of the results in WM studies. Though TBSS has demonstrated not been as much sensitive to ventricular CSF-contamination as other techniques (i.e., WM with voxel-based morphometry)^54^, the elimination of FW in interstitial spaces allows discriminating inflammation-related neuropathy from axonal degeneration^55^. Thus, this correction should be well-considered as it is gaining in presence in recent TBSS studies^56,57^. We have submitted our results to this correction which revealed a substantial contribution of extracellular water content, a surrogate of sustained inflammatory responses potentially prompting axonal degeneration.

On another note, studies tend to explore the effects of stress biomarkers separately. This kind of focus might be not accurate because when stressed, the organism works synergistically. Something similar often occurs when testing the effects that an increase in BMI or WC has on the brain aside from any other physiological variables. In one study, the significant negative relationship between BMI and FA in the fornix and corpus callosum was lost after controlling for cardiovascular and inflammatory factors^58^. This could suggest that the brain abnormalities described in the literature are perhaps associated not to body-weight status or body mass increase as such, but rather to the joint effects of different physiological alterations (e.g., neuroendocrine, immunological, metabolic, and cardiovascular deregulations). In a recent work, we did show that normal-weight and overweight participants respectively exhibited a pattern of cortical thickening and thinning as the AL index increased, regardless of the effects of abdominal obesity^15^. In the current study, normal-weight and overweight subjects showed correlations in the same direction, but only overweight showed a significant relationship between the AL and WM microstructure. According to our hypothesis, overweight participants endure a more significant amount of stress and present greater body fat mass than normal-weight adults, which could explain such outcomes (e.g., obesity-induced low-grade chronic inflammation states). Likewise, the WM could also be more vulnerable to the cardiometabolic comorbidities (i.e., hypertension, hyperlipidaemia, hyperinsulinemia) linked to chronic stress and obesity^23,36,47^.

In another vein, the overweight group showed regional cortical volume reductions in the presence of higher AL index. Concretely, changes in GM density were found in the left precentral gyrus extended to the left postcentral gyrus and the left pars opercularis. Symmetrically, a cluster in the right hemisphere involving the precentral gyrus, the pars opercularis/triangularis, the lateral orbitofrontal gyrus, and the rostral middle frontal gyrus showed a volume decrement as well. Finally, GM alterations in the left lateral occipital gyrus were also found. Frontal and dorsolateral prefrontal areas are mainly known for their role in supervising goal-directed behaviours. Alterations in prefrontal regions and impairments in executive functions have been both described in overweight^59–61^ and stress^45,62,63^. Additionally, some of these areas (i.e., pars opercularis and lateral orbitofrontal gyrus) are also typically known for being involved in the sensorial and hedonic integration of food properties^64,65^. Although scarce or controversial, findings in occipital regions, naturally involved in visual processing and integration, are described in the literature as well^61,66^.

These results are in line with our previous study in which overweight subjects showed a widespread pattern of cortical thinning, especially in frontal and prefrontal regions, regarding the AL index increase15. High levels of AL could induce dendritic retraction or neuronal degeneration via oxidative stress and inflammatory responses ultimately affecting the GM morphology. Conversely, lean subjects did not present positive correlations between the AL index and GM concentrations as in our prior work. Loss of participants (N = 7) in this group due to artefacts during DWI acquisition may have reduced our power to capture any statistically meaningful association. Moreover, volume is three-dimensional measure including length, height, and depth, whilst cortical thickness only considers the latter. Volume is also strongly influenced by width (i.e., surface area), being possible that normal-weight participants presenting greater cortical thickness were showing a pattern of increased cortical folding instead. On another note, there is a partial anatomical overlap between TBSS and SBM results. The IFOF structurally connect occipital areas with dorsolateral prefrontal regions such as the inferior frontal gyri. Some of these areas (i.e., left lateral occipital and pars opercularis/triangularis) volumetrically shrunk relative to higher AL indexes in overweight participants. Likewise, the ACR project fibres from mid-brain regions to superior cortical areas, in which volume reductions were also observed (i.e., precentral and postcentral gyri). Hence, though more research is needed, our results could prompt that physiological stress in otherwise healthy overweight adults is related to GM/WM alterations in anatomically interconnected areas.

The main limitation of the current study was our small sample size. DWI artefacts were present in 16% of the eligible sample, which restricted the number of participants included (e.g., twenty-one lean controls instead of 28). We also refer to the cross-sectional design as another limitation. Because of this, inferences upon causality are not feasible with this methodological approach. To this date, it is still unclear whether the increase in body fat mass is a cause or a consequence of chronic-stress^4,18,19^. We encourage further works to disentangle this issue with longitudinal designs. Another limitation is that we did not include measures of early stressful life events or health behaviours like physical exercise, both known for their ability to modify WM composition^32,38,39^. Moreover, this work could benefit from multimodal neuroimaging approaches fully covering the brain changes related to physiological stress increase. Since our results are limited to skeleton-based WM microstructure, we advise cautiously considering our conclusions. Tractography could inform about whole-tract density or morphology. Moreover, either structural or functional connectivity analyses could help in drawing better assumptions relative to how the brain is wired under these circumstances. We hope to cover these technique-related limitations in further works. Our study also presents methodological strengths, such as the strict criteria employed to ensure that the excess of weight was considered independently of psychiatric (e.g., binge-eating disorder), neurological (e.g., WM lesions), or medical comorbidities (e.g., metabolic syndrome). Thus, we would like to highlight that, even in a non-clinical state, a ‘metabolically healthy overweight’ (MHO) status is associated with alterations in neuroendocrine, immunological, metabolic, and cardiovascular systems. In a recent work, MHO subjects proved to be more at risk for cardiovascular diseases than metabolically healthy normal-weight individuals (age range 18–71)^67^. Hence, overweight is related to a ‘wear and tear’ on biological systems that can be harmful to brain structure (e.g., cortical GM volume reductions, disordered WM composition). In addition, the AL concept requires further in-depth study to facilitate its use by the scientific and medical community. Future works should test for individual effects of each altered biological system in larger samples with a scope on prevention. In the same way, identifying the most important protective factors for stress-related comorbidities would help render public healthcare more efficiently.

In conclusion, our study showed that overweight subjects presented a higher AL index when compared to healthy weight controls. Overweight participants showed an altered WM microstructure regarding AL index increase in the bilateral inferior fronto-occipital fasciculus and the right anterior corona radiata. Some of these changes suggested inflammatory-induced neuropathy. Additionally, this group also presented cortical GM volume reductions concerning AL index increment in the left precentral gyrus, the left lateral occipital gyrus, and the right pars opercularis. Our results indicate that, even in the absence of cardiometabolic comorbidities, an overweight status was related to long-term biological changes potentially harmful to the brain.

## Methods

### Subjects

One hundred and twenty-four participants were recruited from public primary care centres belonging to the *Consorci Sanitari de Terrassa*. Inclusion criteria were (1) being older than 20 years old and (2) having a BMI higher than 18.5 kg/m^2^. Following the World’s Health Organization criteria^68^, the overweight group was formed based on a BMI equal to or higher than 25 kg/m^2^. All volunteers underwent a blood extraction in fasting condition (between 8:00 and 8:30 AM), a medical examination, a comprehensive neuropsychological evaluation, and a magnetic resonance imaging (MRI) acquisition of the head. None of the participants presented comorbidities such as neurological, psychiatric (including addictive or eating disorders), cardiometabolic (including metabolic syndrome, criteria fully described in Appendix A1), developmental, or motor-sensorial disorders. The presence of addictive disorders was evaluated by means of the *Structured Clinical Interview for DSM-IV* (SCID-I). Eating disorders were assessed using the *Bulimia Inventory Test of Edinburgh* (BITE, exclusion criteria were set for scores >20)^69^. Anxiety or depression symptoms were explored using the *Hospital Anxiety and Depression Scale* (HADS, exclusion cut-off score set at ≥11)^70,71^. Additionally, we ruled out participants because of acute-infection suspicion (i.e., C-reactive protein levels >10 mg/L). Moreover, we estimated the intelligence quotient (IQ) using the WAIS-III^72^ vocabulary subtest, excluding participants with scores lower than 7 (i.e., IQ estimated below 85). Finally, we also excluded participants whose MRI report (done by an expert neuroradiologist) indicated the presence of WM hyperintensities. Thirty-four subjects (20 overweight and 14 lean participants) declined to undergo the MRI acquisition by pleading claustrophobia or incompatibilities with the magnetic field. Besides, thirty-eight participants met exclusion criteria (19 overweight and 8 lean subjects), presented artefacts during the diffusion-weighted sequence (3 overweight and 7 lean participants), and/or WM hyperintensities (2 overweight participants and 1 lean subject). Thirty-one overweight and 21 lean controls formed the final sample. Fifty participants (29 overweight-to-obese and 21 healthy weight controls) of this final sample were included from previous studies^15,43,59,73–78^. The study has been conducted following the Helsinki Declaration and has been approved by the University of Barcelona’s Institutional Ethics Committee (CBUB) and the Institutional Review Board (IRB 00003099, assurance No.: FWA00004225; http://www.ub.edu/recerca/comissiobioetica.htm). All methods were performed in accordance with the relevant guidelines and regulations. All participants signed written informed consent before entering the study.

### Allostatic Load Index

Fifteen stress biomarkers were used as in previous studies^12–16^. Leptin was additionally selected as a biomarker since it can induce the release of pro-inflammatory cytokines^79^. Cut-off scores were based on a larger healthy lean population (N = 43) from our preceding study^15^ (see characteristics in Appendix A2). Different cut-off scores were set in those biomarkers that presented differences (p < 0.05) regarding sex (e.g., systolic arterial pressure). Participants who fell into the high-risk biomarker percentile (i.e., 75^th^ or 25^th^, in the case of HDL-cholesterol) were coded with a score of “1”. The AL index was the sum of all 15 dichotomous scores (range 0–15). Higher values on this index indicate a greater AL. Participants with missing values had their AL indexes prorated by the number of available biomarkers. The list of biomarkers and their cut-off scores are presented in Table 3.

**Table 3:** Allostatic load cut-off scores for individual biomarkers.

Biomarker	Male	Female	Both
Systolic arterial pressure (mm Hg)	126	116.50	—
Diastolic arterial pressure (mm Hg)	—	—	72.5
Glycated hemoglobin (%)	—	—	5.40
Glucose (mmol/L)	5.04	4.70	—
Creatinine (umol/L)	89	70	—
Cholesterol (mmol/L)	4.20	4.85	—
HDL (mmol/L)	—	—	1.34
LDL (mmol/L)	—	—	3
Triglycerides (mmol/L)	—	—	0.86
C-reactive protein (mg/L)	—	—	0.93
Interleukin-6 (pg/ml)	—	—	1.65
Insulin (pmol/L)	—	—	53.46
Cortisol (nmol/L)	—	—	657.30
Fibrinogen (g/L)	3.35	3.32	—
Leptin (ng/ml)	4.80	20.30	—

mm Hg = millimetres of mercury, cm = centimetres, mmol/L = millimoles per litre, umol/L = micromoles per litre, mg/L = milligrams per litre, pg/ml = picograms per millilitre, nmol/l = nanomoles per litre, g/L = grams per litre, ng/ml = nanograms per millilitre.

### Other variables of interest

Sociodemographic (i.e., age, years of education, sex, professional level, and total income), psychological (i.e., IQ estimation, subjective anxiety, and depressive symptoms), and other behavioural variables (i.e., tobacco smoking or non-pathological alcohol drinking) were analysed using IBM SPSS Statistics (v.23.0).

### Magnetic-resonance imaging acquisition

Overweight (N = 31) and lean (N = 21) participants underwent MRI on a 3 T MAGNETOM Trio (Siemens, Germany), performed at the *Institut d’Investigacions Biomédiques August Pi I Sunyer* (IDIBAPS) at the *Hospital Clínic* in Barcelona. The diffusion-weighted images (DWI) were acquired with the following parameters: repetition time (TR) = 7,700 ms, echo time (TE) = 89 ms, acquisition matrix = 122 × 122, 2 mm isotropic voxel, field of view (FOV) = 244 × 244 mm^2^, diffusion directions = 30, slice thickness = 2 mm, gap distance = 0.6 mm, number of slices = 60, b-values = 0 and 1,000 s/mm^2^, IPAT factor = 2, total scan time = 4:23 minutes. A T1-weighted MPRAGE 3D sequence was acquired as well for registration, EPI distortion correction, and cortical GM morphometry analysis using the following parameters: TR = 2300 ms, TE = 2.98 ms, inversion time = 900 ms, 240 slices, FOV = 256 mm × 256 mm^2^, 1 mm isotropic voxel.

### Diffusion-tensor imaging processing

All image processing was carried out using the FMRIB Software Library (FSL) v.5.0.10 (https://fsl.fmrib.ox.ac.uk/fsl/fslwiki/FSL) and BrainSuite (BS) v.16a1 (http://brainsuite.org/). First, we visually inspected all DWI sequences to exclude subjects presenting artefacts (N = 10). Second, images were skull-stripped and corrected for head motion and eddy currents. Parallel skull stripping and bias-field correction (FAST) were applied to the T1-weighted images. Geometrical distortions (i.e., EPI distortions) of DWI sequences were solved by using a constrained non-rigid registration to each participants’ T1-weighted image^53^, which is a default step from the BS Diffusion Pipeline. BS also rotates the gradients after this registration to optimise tensor fitting in subsequent steps. The diffusion tensor was fitted to each voxel to generate the FA maps with a linear weighted least squares model to appropriate scale data variances^52^. The most representative FA map was selected, and each subject was projected onto this study-specific template to overcome anatomical misalignments^21^. The mean FA skeleton has been generated based on each participants’ FA values with a threshold of >0.2. Since FA is very unspecific to the source driving the changes in WM microstructure, we tested other complementary diffusivity scalars. The MD is the average of all eigenvalues with higher values meaning exacerbated cell permeability, presumably due to oedema or necrosis. The AD and RD reflect parallel and perpendicular diffusion, respectively. Lower AD values tend to be present in contexts of axonal damage, while higher RD values could indicate poor myelination^80^. MD, AD, and RD were also projected onto the mean FA skeleton for complementary analysis. Additionally, FW may alter DTI metrics, and its elimination showed an improvement in tract-reconstruction, tissue segmentation, and characterisation of underlying pathological disturbances^22,81^. Hence, a bi-tensor model was fitted with the algorithm developed by Pasternak *et al*.^81^. Corrected FA maps were projected into the normalised FA skeleton for further *post-hoc* analysis.

### Tract-based spatial statistics

Statistical analyses were performed with non-parametric permutation-based tests (10,000 iterations) correcting all results for multiple comparisons using an FWE procedure and a threshold-free cluster enhancement. Statistical significance was set at FWE corrected p-value < 0.05. Age and sex were selected as nuisance factors in all analyses. Moreover, as we aimed to explore the relationship between AL and WM composition isolated from abdominal obesity, we have regressed out its effects as in our previous work. The WtHR was included in the principal model as an additional nuisance factor. This measure has proved to reflect better than BMI and WC the amount of visceral adipose tissue and its adverse outcomes (i.e., cardiovascular risk)^82,83^. First, we extracted the average diffusivity values for the skeletonised maps and test for differences between groups controlling for age, sex, and AL. Second, we conducted a whole-brain voxel-wise analysis to test for regional differences in diffusivity metrics between groups (nuisance factors: age, sex, and AL). Third, whole-brain correlation analysis with the AL index as the independent variable was conducted, first in the entire group (N = 52) and then between groups (nuisance factors: age, sex, and WtHR) for all DTI metrics. Third, clusters showing a significant relationship with the AL index were fed into a *post-hoc* analysis in FSL randomise (family-wise error corrected at p < 0.05, 10,000 permutations) with the FW corrected maps. We extracted the average diffusivity scores within each significant cluster to plot them against the AL index. We calculated the magnitude (i.e., Pearson’s coefficient) of this relationship for visual purposes only using the FA standardised residual to control the effects of age, sex and WtHR. Note that we did not report the p-values of this correlation as its statistical significance has been already covered in the principal analysis. Since the skeletonised maps are a one-voxel-thick image, significant results were thickened 3 mm to ease their visualisation. The White-Matter Tractography Atlas and the ICBM DTI-81 White-Matter Labels, both from the John Hopkins University, were used to label each cluster according to their peak and extension. We additionally controlled the effects of tobacco smoking and non-pathological alcohol drinking, considering this as a complementary analysis due to the high risk of overfitting given our small sample size. Moreover, as the AL and the abdominal obesity increase are intrinsically related, we ran the same analysis only regressing out the effects of age and sex. The results of these two analyses are available with more detail in the SI section.

### Grey matter morphometry analysis

Parallel to the principal analysis on TBSS, we have also tested for cortical GM volume (mm^3^) changes in FreeSurfer v.6.0 (https://surfer.nmr.mgh.harvard.edu) with a surface-based approach. Surface-based morphometry (SBM) allows a better characterisation of the GM tissue as it considers the complicated topology of the cortical surface (i.e., gyri, sulci). Traditional voxel-based morphometry (VBM) approaches are limited in considering the intricate composition of a highly folded structure such as the human brain cortex is. VBM uses a probabilistic tissue segmentation approach where each voxel is classified correspondingly to the proportion of contained tissue (i.e., GM, WM, and CSF). In contrast, SBM reconstructs the cortical surface using the boundaries of GM and WM layers in a convoluted triangulated mesh. Thus, T1-weighted images have been processed following this approach using the cortical reconstruction pipeline in FreeSurfer. Briefly, this process includes motion and intensity correction, removal of the skull and soft tissue, normalisation, segmentation, parcellation, and smoothing^84–86^. We have followed the same model as in the principal analysis in TBSS, where the AL index was the variable of interest and sex, age, and WtHR were selected as nuisance factors. The SBM analysis was performed with the Query, Design, Estimate, Contrast (Qdec) tool implemented in FreeSurfer. The surface was smoothed using a circularly symmetric Gaussian kernel with a full-width at half-maximum smoothing of 15 mm. We have also controlled for multiple comparisons using a Monte-Carlo null-Z Simulation (10,000 repetitions) with a cluster-wise correction. Cortical structures were named after the Desikan atlas^87^, and cluster coordinates were reported accordingly to MNI space. We calculated the mean GM volume (mm^3^) of significant clusters and controlled the effects of age, sex, and WtHR to plot them against the AL index. Again, the reported Pearson’s coefficients were only to facilitate visual interpretation.

## Electronic supplementary material

Supplementary Information

## Data Availability

The datasets analysed during the current study are available from the corresponding author on reasonable request.

## References

[1] Berthoud H-R, Morrison C (2008). The Brain, Appetite, and Obesity. Annu. Rev. Psychol..

[2] Reilly SM, Saltiel AR (2017). Adapting to obesity with adipose tissue inflammation. Nat. Rev. Endocrinol..

[3] Guillemot-Legris O, Muccioli GG (2017). Obesity-Induced Neuroinflammation: Beyond the Hypothalamus. Trends Neurosci..

[4] Foss B, Dyrstad SM (2011). Stress in obesity: Cause or consequence?. Med. Hypotheses.

[5] Arnsten AFT (2009). Stress signalling pathways that impair prefrontal cortex structure and function. Nat. Rev. Neurosci..

[6] McEwen BS, Nasca C, Gray JD (2016). Stress Effects on Neuronal Structure: Hippocampus, Amygdala and Prefrontal Cortex. Neuropsychopharmacology.

[7] Groppe K, Elsner B (2015). The influence of hot and cool executive function on the development of eating styles related to overweight in children. Appetite.

[8] McEwen BS, Stellar E (1993). Stress and the individual. Mechanisms leading to disease. Arch. Intern. Med..

[9] Juster RP, McEwen BS, Lupien SJ (2010). Allostatic load biomarkers of chronic stress and impact on health and cognition. Neurosci. Biobehav. Rev..

[10] Calcia MA (2016). Stress and neuroinflammation: a systematic review of the effects of stress on microglia and the implications for mental illness. Psychopharmacology (Berl)..

[11] Jauregui-Huerta F (2010). Responses of Glial Cells to Stress and Glucocorticoids. Curr. Immunol. Rev..

[12] Booth T (2015). Association of allostatic load with brain structure and cognitive ability in later life. Neurobiol. Aging.

[13] Chiappelli J (2017). Allostatic load and reduced cortical thickness in schizophrenia. Psychoneuroendocrinology.

[14] Savransky A (2017). Fornix Structural Connectivity and Allostatic Load. Psychosom. Med..

[15] Ottino-González J (2017). Allostatic Load Is Linked to Cortical Thickness Changes Depending on Body-Weight Status. Front. Hum. Neurosci..

[16] Cole JH (2017). Brain age predicts mortality. Mol. Psychiatry.

[17] Jackson SE, Kirschbaum C, Steptoe A (2017). Hair cortisol and adiposity in a population-based sample of 2,527 men and women aged 54 to 87 years. Obesity.

[18] Sinha R, Jastreboff AM (2013). Stress as a Common Risk Factor for Obesity and Addiction. Biol. Psychiatry.

[19] Sominsky L, Spencer SJ (2014). Eating behavior and stress: a pathway to obesity. Front. Psychol..

[20] Basser PJ, Mattiello J (1994). & Lebihan, D. MR Diffusion Tensor Spectroscopy and Imaging. Biophys. J..

[21] Bach M (2014). Methodological considerations on tract-based spatial statistics (TBSS). Neuroimage.

[22] Pasternak O (2012). Excessive Extracellular Volume Reveals a Neurodegenerative Pattern in Schizophrenia Onset. J. Neurosci..

[23] Kullmann S, Schweizer F, Veit R, Fritsche A, Preissl H (2015). Compromised white matter integrity in obesity. Obes. Rev..

[24] Kullmann S (2016). Specific white matter tissue microstructure changes associated with obesity. Neuroimage.

[25] Papageorgiou I (2017). Abnormalities of brain neural circuits related to obesity: A Diffusion Tensor Imaging study. Magn. Reson. Imaging.

[26] Birdsill AC (2017). Abdominal obesity and white matter microstructure in midlife. Hum. Brain Mapp..

[27] Ou X, Andres A, Pivik RT, Cleves MA, Badger TM (2015). Brain gray and white matter differences in healthy normal weight and obese children. J. Magn. Reson. Imaging.

[28] Stanek KM (2010). Obesity Is Associated With Reduced White Matter Integrity in Otherwise Healthy Adults. Obesity (Silver Spring)..

[29] Ryan L, Walther K (2014). White matter integrity in older females is altered by increased body fat. Obesity.

[30] Zhang R (2018). White matter microstructural variability mediates the relation between obesity and cognition in healthy adults. Neuroimage.

[31] Repple J (2018). Elevated body-mass index is associated with reduced white matter integrity in two large independent cohorts. Psychoneuroendocrinology.

[32] Sheikh HI (2014). Links between white matter microstructure and cortisol reactivity to stress in early childhood: Evidence for moderation by parenting. NeuroImage Clin..

[33] van der Werff SJA (2014). Widespread reductions of white matter integrity in patients with long-term remission of Cushing’s disease. NeuroImage Clin..

[34] Nugent KL (2015). Cortisol Reactivity to Stress and Its Association With White Matter Integrity in Adults With Schizophrenia. Psychosom. Med..

[35] Cohen JI, Cazettes F, Convit A (2011). Abnormal cholesterol is associated with prefrontal white matter abnormalities among obese adults, a diffusion tensor imaging study. Neuroradiol. J..

[36] Maillard P (2012). Effects of systolic blood pressure on white-matter integrity in young adults in the Framingham Heart Study: a cross-sectional study. Lancet Neurol..

[37] Bettcher BM (2015). Declines in inflammation predict greater white matter microstructure in older adults. Neurobiol. Aging.

[38] Schaeffer DJ (2014). An 8-month exercise intervention alters frontotemporal white matter integrity in overweight children. Psychophysiology.

[39] Voss MW (2013). The influence of aerobic fitness on cerebral white matter integrity and cognitive function in older adults: Results of a one-year exercise intervention. Hum. Brain Mapp..

[40] Koch K (2014). Association between white matter fiber structure and reward-related reactivity of the ventral striatum. Hum. Brain Mapp..

[41] Gianaros PJ, Marsland AL, Sheu LK, Erickson KI, Verstynen TD (2013). Inflammatory pathways link socioeconomic inequalities to white matter architecture. Cereb. Cortex.

[42] Starcke K, Brand M (2016). Effects of stress on decisions under uncertainty: A meta-analysis. Psychol. Bull..

[43] Marqués-Iturria I (2015). Affected connectivity organization of the reward system structure in obesity. Neuroimage.

[44] Higgs S (2016). Cognitive processing of food rewards. Appetite.

[45] Sandi C (2013). Stress and cognition. Wiley Interdiscip. Rev. Cogn. Sci..

[46] Alarcón G, Ray S, Nagel BJ (2016). Lower Working Memory Performance in Overweight and Obese Adolescents Is Mediated by White Matter Microstructure HHS Public Access. J Int Neuropsychol Soc. J Int Neuropsychol Soc.

[47] Lundgaard I, Osório MJ, Kress BT, Sanggaard S, Nedergaard M (2014). White matter astrocytes in health and disease. Neuroscience.

[48] Qiu L (2014). Regional increases of cortical thickness in untreated, first-episode major depressive disorder. Transl. Psychiatry.

[49] Kim Y, Kim Y, Won E (2017). The influence of stress on neuroinflammation and alterations in brain structure and function in major depressive disorder structure and function in major depressive disorder. Behav. Brain Res..

[50] Zatorre RJ, Fields RD, Johansen-Berg H (2012). Plasticity in gray and white: neuroimaging changes in brain structure during learning. Nat. Neurosci..

[51] Streit WJ (2006). Microglial senescence: does the brain’s immune system have an expiration date?. Trends Neurosci..

[52] Jones DK, Knösche TR, Turner R (2013). White matter integrity, fiber count, and other fallacies: The do’s and don’ts of diffusion MRI. Neuroimage.

[53] Peper Jiska S., Dahl Ronald E. (2013). The Teenage Brain. Current Directions in Psychological Science.

[54] Bergamino M, Kuplicki R, Victor TA, Cha Y-H, Paulus MP (2017). Comparison of two different analysis approaches for DTI free-water corrected and uncorrected maps in the study of white matter microstructural integrity in individuals with depression. Hum. Brain Mapp..

[55] Bergamino M, Pasternak O, Farmer M, Shenton ME, Paul Hamilton J (2016). Applying a free-water correction to diffusion imaging data uncovers stress-related neural pathology in depression. NeuroImage Clin..

[56] Guttuso T (2018). Substantia Nigra Free Water Increases Longitudinally in Parkinson Disease. Am. J. Neuroradiol..

[57] Kaufmann LK (2017). Fornix Under Water? Ventricular Enlargement Biases Forniceal Diffusion Magnetic Resonance Imaging Indices in Anorexia Nervosa. Biol. Psychiatry Cogn. Neurosci. Neuroimaging.

[58] Bettcher BM (2013). Body Mass and White Matter Integrity: The Influence of Vascular and Inflammatory Markers. PLoS One.

[59] Marqués-Iturria I (2013). Frontal cortical thinning and subcortical volume reductions in early adulthood obesity. Psychiatry Res. Neuroimaging.

[60] Smith E, Hay P, Campbell L, Trollor JN (2011). A review of the association between obesity and cognitive function across the lifespan: implications for novel approaches to prevention and treatment. Obes. Rev..

[61] Veit R (2014). Reduced cortical thickness associated with visceral fat and BMI. NeuroImage. Clin..

[62] Savic I (2015). Structural Changes of the Brain in Relation to Occupational Stress. Cereb. Cortex.

[63] Zhang H (2016). The relationship between inflammatory markers and voxel-based gray matter volumes in nondemented older adults. Neurobiol. Aging.

[64] Kumar S (2016). Differences in Insula and Pre-/Frontal Responses during Reappraisal of Food in Lean and Obese Humans. Front. Hum. Neurosci..

[65] Small DM (2007). The role of the human orbitofrontal cortex in taste and flavor processing. Ann. N. Y. Acad. Sci..

[66] Willette Auriel A., Kapogiannis Dimitrios (2015). Does the brain shrink as the waist expands?. Ageing Research Reviews.

[67] Caleyachetty R (2017). Metabolically Healthy Obese and Incident Cardiovascular Disease Events Among 3.5 Million Men and Women. J. Am. Coll. Cardiol..

[68] World Health Organization. Obesity and overweight. *WHO* (2016). Available at: http://www.who.int/mediacentre/factsheets/fs311/en/. (Accessed: 16th December 2016).

[69] Henderson M, Freeman CP (1987). A self-rating scale for bulimia. The ‘BITE’. Br. J. Psychiatry.

[70] Zigmond AS, Snaith RP (1983). The Hospital Anxiety and Depression Scale. Acta Psychiatr. Scand..

[71] Herrero MJ (2003). A validation study of the hospital anxiety and depression scale (HADS) in a Spanish population. Gen. Hosp. Psychiatry.

[72] Wechsler, D. *WAIS III. Escala de Inteligencia de Wechsler para adultos III (adaptación española ed*.). (TEA Editores S.A., 1999).

[73] García-García I. (2013). Functional connectivity in obesity during reward processing. NeuroImage.

[74] García-García Isabel (2013). Alterations of the salience network in obesity: A resting-state fMRI study. Human Brain Mapping.

[75] García-García I. (2013). Neural Responses to Visual Food Cues: Insights from Functional Magnetic Resonance Imaging. European Eating Disorders Review.

[76] García-García Isabel (2015). Functional network centrality in obesity: A resting-state and task fMRI study. Psychiatry Research: Neuroimaging.

[77] Ariza Mar (2012). Dopamine Genes (DRD2/ANKK1-TaqA1 and DRD4-7R) and Executive Function: Their Interaction with Obesity. PLoS ONE.

[78] Marqués-Iturria Idoia (2014). The interaction effect between BDNFpolymorphism and obesity on executive functions and frontal structure. American Journal of Medical Genetics Part B: Neuropsychiatric Genetics.

[79] Abella V (2017). Leptin in the interplay of inflammation, metabolism and immune system disorders. Nat. Rev. Rheumatol..

[80] Alexander AL (2011). Characterization of Cerebral White Matter Properties Using Quantitative Magnetic Resonance Imaging Stains. Brain Connect..

[81] Pasternak O, Sochen N, Gur Y, Intrator N, Assaf Y (2009). Free water elimination and mapping from diffusion MRI. Magn. Reson. Med..

[82] Swainson MG, Batterham AM, Tsakirides C, Rutherford ZH, Hind K (2017). Prediction of whole-body fat percentage and visceral adipose tissue mass from five anthropometric variables. PLoS One.

[83] Ashwell M, Gibson S (2016). Waist-to-height ratio as an indicator of ‘early health risk’: simpler and more predictive than using a ‘matrix’ based on BMI and waist circumference. BMJ Open.

[84] Ségonne F (2004). A hybrid approach to the skull stripping problem in MRI. Neuroimage.

[85] Reuter M, Rosas HD, Fischl B (2010). Highly accurate inverse consistent registration: A robust approach. Neuroimage.

[86] Sled JG, Zijdenbos AP, Evans AC (1998). A nonparametric method for automatic correction of intensity nonuniformity in MRI data. IEEE Trans. Med. Imaging.

[87] Desikan RS (2006). An automated labeling system for subdividing the human cerebral cortex on MRI scans into gyral based regions of interest. Neuroimage.

