# Supplementary material for: Allostatic load and disordered white matter microstructure in overweight adults
Source: Sci Rep. 2018 Oct 26;8:15898. doi: 10.1038/s41598-018-34219-8 (PMC6203765; doi:10.1038/s41598-018-34219-8)
Supplement: Supplementary file 1 — Supplementary Information [file 41598_2018_34219_MOESM1_ESM.docx]

**Title:** Allostatic load and disordered white matter microstructure in overweight adults

**Authors:** Ottino-González J^a,c,d^, **Jurado MA(*)^a,c,d^**, García-García I^i^, Segura B^b,e^, Marqués-Iturria I^a^, Sender-Palacios MJ^f,h^, Tor E^f,h^, Prats-Soteras X^a,c,d^, Caldú X^a,c,d^, Junqué C^b,c,e^, Pasternak O^j^, Garolera M^g,h^ .

1. **Supplementary Information**

Appendix A1 – Metabolic syndrome criteria (Alberti et al., 2009). Participants should meet at least three of the following criteria:

1. Elevated waist circumference (≥ 94 centimetres for males, ≥ 80 centimetres for females)
2. Elevated triglycerides (≥ 150 mg/dL)
3. Low high-density lipoprotein (HDL) cholesterol (≤ 40 mg/dL in males, ≤ 50 mg/dL in females)
4. High arterial pressure (≥ 130 mm Hg systolic and/or ≥ 85 mm Hg diastolic arterial pressure)
5. Elevated fasting glucose (≥ 100 mg/dL)

mg = milligrams, dL = decilitres, mm = millimetres, Hg = mercury

**Appendix A2 – Characteristics of the normative group (cut-off calculation)**

|  | **Lean (N = 43)** | |
| --- | --- | --- |
|  | **Mean (SD)** | **Range** |
| Age | 30.44 (6.03) | 21 – 40 |
| Years of education | 14.12 (2.41) | 9 – 18 |
| IQ estimation | 11.50 (1.95) | 7 – 15 |
| Female N (%) | 26 (60.5%) | |
| Smoker N (%) | 9 (20.9%) | |
| Drinker N (%) | 24 (55.8%) | |
| HADS anxiety | 4.47 (2.80) | 0 – 10 |
| HADS depression | 1.26 (1.60) | 0 - 6 |
| BMI | 21.99 (1.76) | 18.59 – 24.99 |
| WC | 76.05 (6.95) | 61 – 92 |
| WtHR | 0.45 (0.03) | 0.40 – 0.51 |
| **Frequency of family income in euros per month (%)** | | |
| 300 - 899 | 1 (2.3%) | |
| 900 - 1,499 | 7 (16.3%) | |
| 1,500 - 2,099 | 13 (30.2%) | |
| 2,100 - 2,699 | 7 (16.3%) | |
| > 2,700 | 13 (30.2%) | |
| Not available | 2 (4.7%) | |
| **Frequency of professional level (%)** | | |
| Non-skilled | 4 (9.3%) | |
| Skilled manual | 5 (11.6%) | |
| Administrative | 8 (18.6%) | |
| Intermediate | 7 (16.3%) | |
| Professional | 9 (20.9%) | |
| Not available | 10 (23.3%) | |
| IQ estimation = intelligence quotient estimation (WAIS-III vocabulary scalar score), BMI = body mass index (kg/m^2^), WC = waist circumference (centimetres), WtHR = waist-to-height ratio (WC/height in centimetres), HADS = Hospital Anxiety and Depression Scale, SD = standard deviation. | | |

**Appendix B1 – ANOVA for global FA, MD, AD and RD skeleton maps**


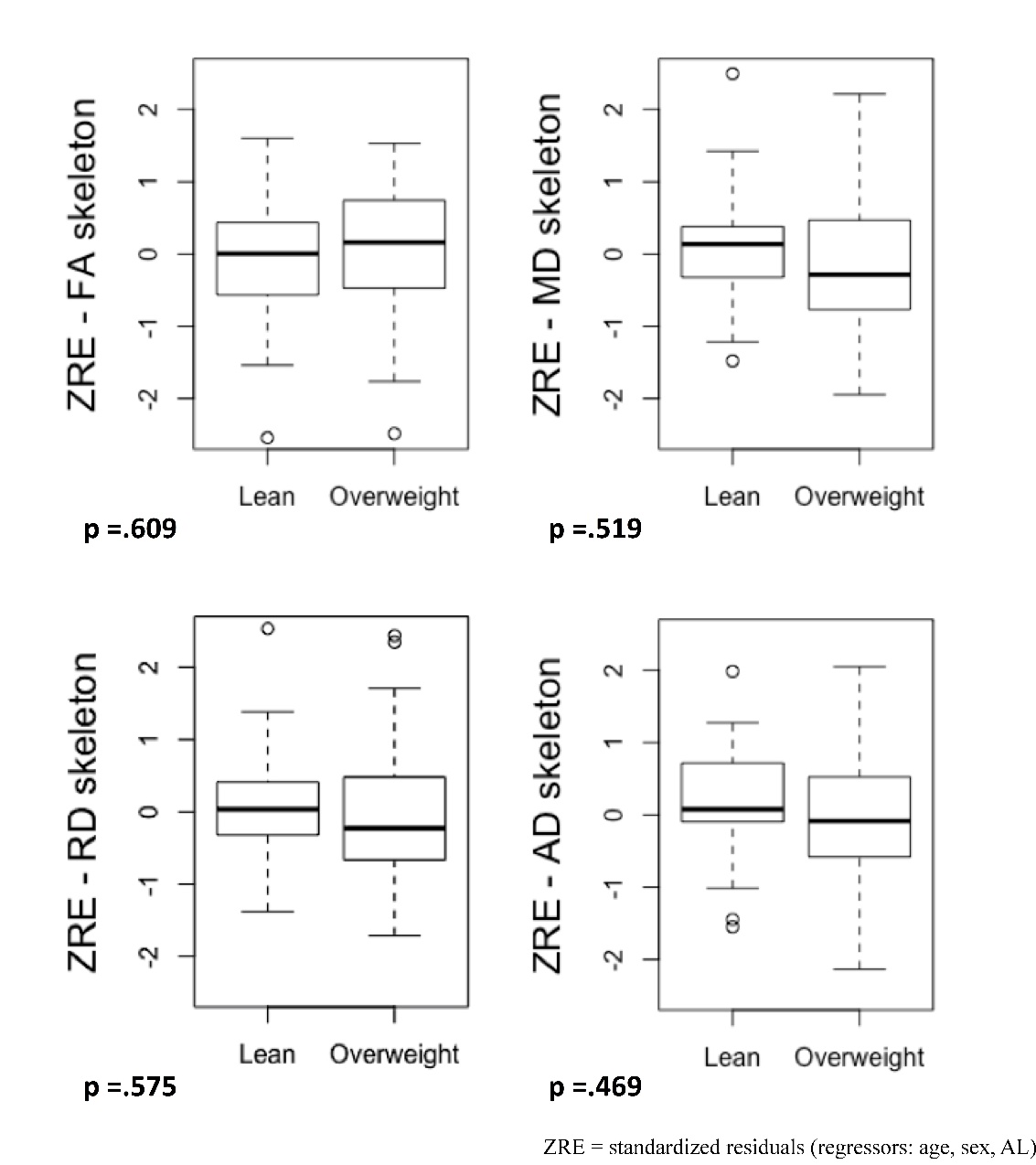


ZRE = standardised residuals (regressors: age, sex, and AL)

**Appendix B2 – Whole-brain correlations in the overweight group (additionally controlling for tobacco/alcohol usage)**

|  | **Peak** | **Voxels** | **MNI**  **Coordinates** | | | **Cluster extension** | **FWE**  **p-value** |
| --- | --- | --- | --- | --- | --- | --- | --- |
|  |  |  | **X** | **Y** | **Z** |  |  |
| **FA** | **L IFOF** | 845 | -32 | -55 | 15 | L anterior/posterior thalamic radiation, L cingulum (hippocampus), forceps major, splenium of corpus callosum, L inferior fronto-occipital fasciculus, L inferior longitudinal fasciculus, L superior longitudinal fasciculus (regular and temporal part). | 0.035 |
|  | **R IFOF** | 78 | 31 | -65 | 1 | R anterior/posterior thalamic radiation, R cingulum (hippocampus), forceps major, R inferior fronto-occipital fasciculus, R inferior longitudinal fasciculus. | 0.047 |
|  | **Body of CC** | 481 | -2 | 11 | 22 | R anterior thalamic radiation, R cingulum (cingulate gyrus), forceps minor, genu and body of corpus callosum, R superior longitudinal fasciculus (regular and temporal part). | 0.038 |
|  | **R ACR** | 88 | 18 | 21 | 34 | R anterior thalamic radiation, body of corpus callosum, R anterior/superior corona radiata, R cingulum (cingulate gyrus), R superior longitudinal fasciculus (regular and temporal part). | 0.048 |
| MNI = Montreal Neurological Institute, FWE = family-wise error, FA = fractional anisotropy, L IFOF = left inferior fronto-occipital fasciculus, R IFOF = right inferior fronto-occipital fasciculus, CC = corpus callosum, R ACR = right anterior corona radiata | | | | | | | |

**
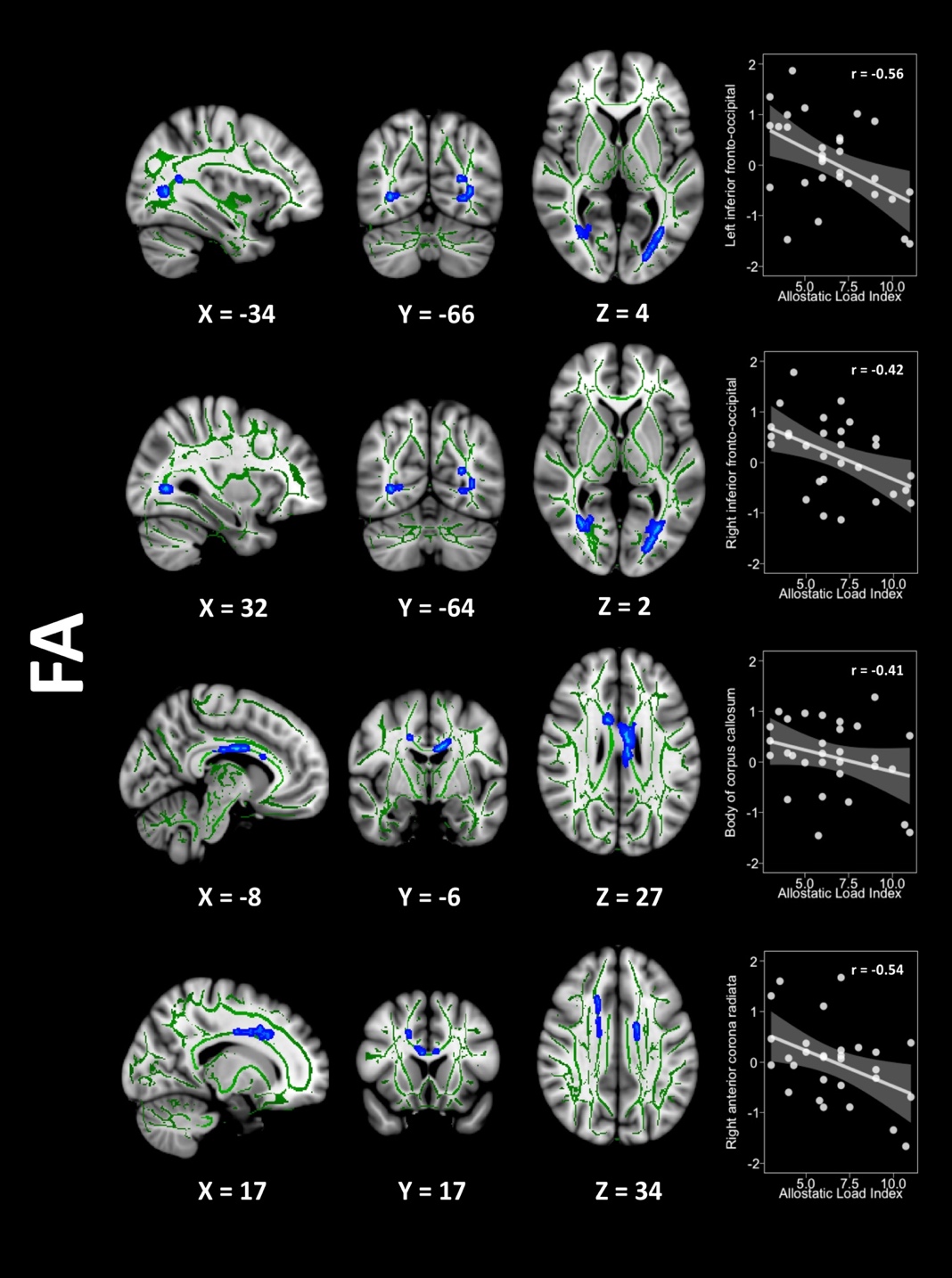
**

Appendix C1 – The first four rows show in blue the location and extension of the decreasing FA values regarding the AL index increase. The Y-axis in the scatterplots depicts the average diffusivity standardised FA score within the cluster after controlling for age, sex, WtHR, tobacco, and alcohol usage. The X-axis represents the AL index scores. Please note that correlation coefficients (r) only intend to complement scatterplots visualization. FA = fractional anisotropy, WtHR = waist-to-height ratio.

**Appendix C2 – Whole-brain correlations in the overweight group (not controlling for WtHR)**

|  | **Peak** | **Voxels** | **MNI**  **Coordinates** | | | **Cluster extension** | **FWE**  **p-value** |
| --- | --- | --- | --- | --- | --- | --- | --- |
|  |  |  | **X** | **Y** | **Z** |  |  |
| **FA** | **R ACR** | 2812 | 17 | 15 | 35 | Body and splenium of corpus callosum, R uncinate, R superior and inferior fronto-occipital fasciculus, R external and posterior limb of the internal capsule, R anterior, superior and posterior corona radiata, R corticospinal tract, R anterior thalamic radiation and L superior corona radiata. | 0.025 |
|  | **R internal capsule** | 55 | 21 | 12 | 121 | R anterior limb of the internal capsule, R anterior thalamic radiation, R forceps minor, R anterior corona radiata and R inferior fronto-occipital fasiculus | 0.043 |
|  | **L ACR** | 273 | -16 | 20 | 30 | L anterior and superior corona radiata, L anterior thalamic radiation, L superior longitudinal fasciculus, L cingulum and L forceps minor | 0.039 |
| **RD** | **R PCR** | 79 | 17 | 0 | 36 | R superior and anterior corona radiata and body of corpus callosum | 0.048 |
| MNI = Montreal Neurological Institute, FWE = family-wise error, FA = fractional anisotropy, RD = radial diffusivity, R ACR = right anterior corona radiata, L ACR = left anterior corona radiata, R PCR = right posterior corona radiata | | | | | | | |


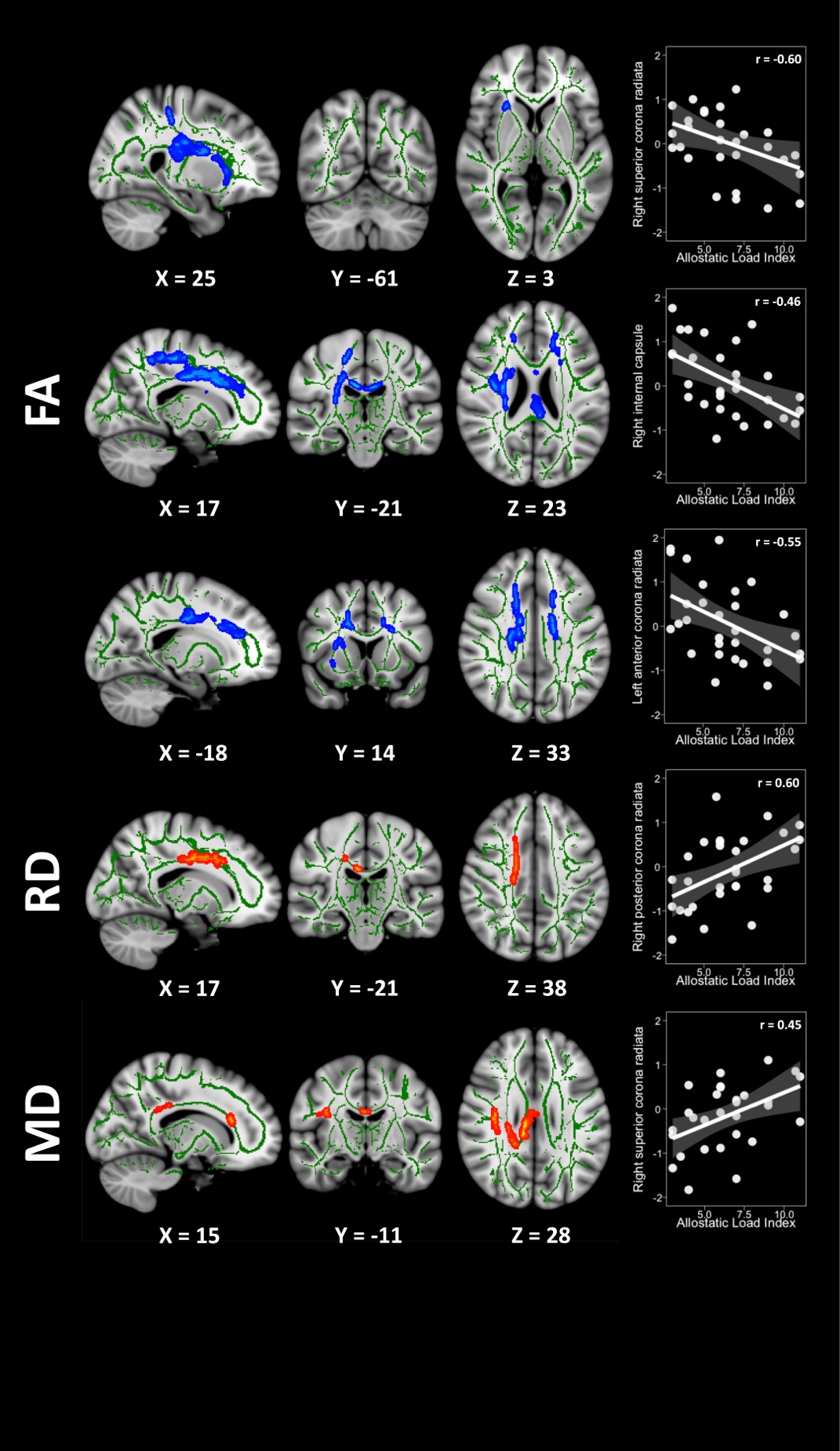


Appendix D1 – The first three rows show in blue the location and extension of the decreasing FA values regarding the AL index increase. The fourth row shows in red the relationship between RD and AL index. The Y-axis in the scatterplots depicts the average diffusivity standardised FA score within the cluster after controlling for age and sex. The X-axis represents the AL index scores. Please note that correlation coefficients (r) only intend to complement scatterplots visualization. FA = fractional anisotropy, RD = radial diffusivity.
